# Plasmonic Fiber Optic Refractometric Sensors: From Conventional Architectures to Recent Design Trends

**DOI:** 10.3390/s17010012

**Published:** 2016-12-23

**Authors:** Elizaveta Klantsataya, Peipei Jia, Heike Ebendorff-Heidepriem, Tanya M. Monro, Alexandre François

**Affiliations:** 1 Institute for Photonics and Advanced Sensing, The University of Adelaide, Adelaide, SA 5005, Australia; peipei.jia@adelaide.edu.au (P.J.); heike.ebendorff@adelaide.edu.au (H.E.-H.); tanya.monro@unisa.edu.au (T.M.M.); alexandre.francois@unisa.edu.au (A.F.); 2 ARC Centre of Excellence for Nanoscale BioPhotonics (CNBP), The University of Adelaide, Adelaide, SA 5005, Australia; 3 The University of South Australia, Adelaide, SA 5001, Australia

**Keywords:** fiber sensors, Surface Plasmon Resonance (SPR), plasmonics, optical fiber, Localized Surface Plasmon Resonance (LSPR)

## Abstract

Surface Plasmon Resonance (SPR) fiber sensor research has grown since the first demonstration over 20 year ago into a rich and diverse field with a wide range of optical fiber architectures, plasmonic coatings, and excitation and interrogation methods. Yet, the large diversity of SPR fiber sensor designs has made it difficult to understand the advantages of each approach. Here, we review SPR fiber sensor architectures, covering the latest developments from optical fiber geometries to plasmonic coatings. By developing a systematic approach to fiber-based SPR designs, we identify and discuss future research opportunities based on a performance comparison of the different approaches for sensing applications.

## 1. Introduction

Plasmonics is a well-established research field which has been extensively studied over the last few decades. Since the pioneering work by Kretschmann et al. [1] and Otto [2] in the late sixties, who demonstrated optical excitation of Surface Plasmons (SPs) by means of attenuated total reflection using a prism, a wide range of sensing architectures has emerged [3]. SPs are collective oscillations of the free electron gas in metals that exist at the interface between two materials with dielectric constants of opposite sign, such as a metal and a dielectric [4]. When the propagation constant of the excitation photons matches that of the electron oscillations, Surface Plasmon Resonance (SPR) occurs, where part of the energy of the incident light is transferred to the SPs. The resonant condition depends on the dielectric constants of the metal and dielectric that support the interface, but also on the refractive index of the medium within the SP evanescent field. The later has been first exploited by Pockrand et al. [5] and Liedberg et al. [6], who both used the change of a resonant condition for monitoring binding events onto a silver thin film, thereby creating the first optical label-free biosensing platform.

Commercial SPR systems are now a reality, with several manufacturers still exploiting the Kretschmann prism configuration in their instruments. However, substituting the prism excitation scheme with optical fibers has rapidly emerged since the early 90s, taking advantage of the cost effectiveness and miniaturization potential associated with optical fibers [7,8]. This early work on SPR fiber sensors paved the way for further development of the fiber geometries, interrogation methods and plasmonic coatings, allowing for improved sensing performance and alternative sensing modalities. The emergence of fiber-based SPR sensors created unique research and commercial opportunities, such as in vivo medical diagnostic [9], or the development of point-of-care devices using smartphone technologies [10,11].

However, due to the large diversity of SPR fiber sensor designs, it is difficult to understand the pros and cons of each SPR fiber architecture. Although, several reviews on fiber-based SPR sensors have previously been published [12,13,14,15], none presented a complete comparison of the different designs encompassing optical fiber and metallic coatings, SPR excitation and interrogation methods, and sensing applications. Therefore, the objective of this review article is to provide a comprehensive comparison of the performance of the state of the art SPR fiber sensors based on the different technological approaches. The following sections describe the different SPR interrogation methods and the resulting operating principles. Various SPR fiber designs from both, the optical fiber architecture and the metallic coating perspectives, are also discussed, along with examples of applications. Finally, a comparison between the different architectures is provided based on their respective performance, followed by a discussion on future research prospects in this field.

## 2. Operating Principles of Plasmonic Fiber Sensors

SPs can be viewed as an electromagnetic wave that propagates along the interface between metal and dielectric. Electromagnetic field associated with SPs at the resonance reaches maximum at the interface and exponentially decays into both metal and dielectric. The penetration depth and propagation length of the plasmons are determined by the dielectric constant of metal and its thickness, as well as the refractive index of the adjacent dielectric. This enhanced electromagnetic field in the dielectric medium and strong dependency of the resonance condition on the optical properties of the supporting interface make SPR an indispensable tool for refractive index sensing.

The fundamental principle of SP excitation in optical fibers is similar to the prism-based techniques, known as Kretschmann [1] and Otto [2] configurations. Propagating SPs cannot be excited by light incident on the metal film from air, therefore, a dielectric with higher refractive index (such as a prism or a fiber core) is used to enhance the propagation constant of the incident light [4]. For excitation of SPs to occur, the propagation constant of the incident light must match that of the SPs (*k_SP_*):

(1) $$k_{SP}=k_{0}{\left(\frac{\varepsilon_{m}\varepsilon_{s}}{\varepsilon_{m}+\varepsilon_{s}}\right)}^{1/2}$$

where *ε_m_* = Re(*ε_m_*) + i*Im*(*ε_m_*) and *ε_m_* and *ε_s_* are the dielectric constants of the metal and dielectric, respectively; and *k*_0_ = *ω*/*c* is the free-space wave number, where *ω* is the angular frequency of the incident light, and *c* is the speed of light in vacuum. Equation (1) describes a guided mode (SP wave) only if Re(*ε_m_*) < −Re(*ε_s_*). This condition is, in general, fulfilled for a metal and dielectric interface [4]. The component of the wave propagation constant parallel to the metal-dielectric interface is given by:

(2) $$k=k_{0}\sqrt{\varepsilon_{p}}\sin\left(\theta \right)$$

where *ε_p_* is the dielectric constant of the prism. To satisfy the coupling condition *k* = *k_sp_*, the incidence angle (*θ*) can be adjusted. However, for an optical fiber, the propagation constant of the guides modes is determined by the fiber properties (i.e., core diameter, core and cladding refractive indices), and cannot be changed dynamically. While the fiber-based approach imposes some inherent restrictions compared with the prism coupling scheme, a range of SPR excitation methods have been developed for optical fibers, enabled by the diversity of optical fiber architectures, which is discussed in Section 3.1.

### 2.1. Interrogation Methods

Most of the SPR interrogation methods in optical fibre are based on the detection of loss in the transmitted light at the resonance, associated with coupling of the guided fibre modes to SP modes. This is achieved through a large variety of methods ranging from the spectral interrogation of transmitted [7,16,17,18,19,20] or back reflected [21,22,23] signal using a broadband excitation source, to the measurement of intensity near resonance [10,23,24,25,26]. Also, analyses of phase changes due to SPR have been reported [27,28].

#### 2.1.1. Spectral Interrogation

**Figure 1:**
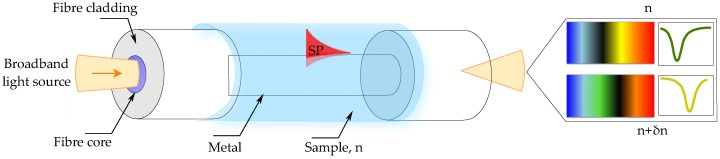
Fiber-based SPR sensor. Change in the refractive index of the sample (δn) causes change in the resonant condition, which is seen as a shift of the resonant wavelength (δλ) (dip in the transmitted spectra).

Spectral interrogation is by far the most commonly employed technique for SPR fiber sensors [16,17,18,19,20]. This configuration was first proposed by Jorgenson et al. [7], and is depicted in Figure 1. As the guided white light interacts with a metallic coating deposited onto the fiber core, the transmitted light is being depleted at specific wavelengths, corresponding to the optical modes whose propagation constants match that of the SPs. Spectral interrogation can be achieved either in transmission or in back-reflection measurements, where in the later configuration the light propagating inside the fiber reflects back from a mirror deposited on the fiber tip, allowing for dip sensor architectures [21,22,23]. The advantage of the back reflection technique is that the analyzed light undergoes interaction with the sensing region twice [21]. However, it increases the complexity of the required optical setup when compared with conventional transmission measurements. A choice between transmission or back reflection measurements is typically dictated by the application.

#### 2.1.2. Intensity Interrogation

A monochromatic light source can be used to excite SPs, providing that the wavelength of excitation is chosen such that the combination of the wavelength and incidence angles satisfies the resonant condition. SPR is observed indirectly through analyzing the intensity of the collected light, either transmitted or back-reflected [10,23,24,25,26]. The advantage of this technique is its simplicity and low cost. However, careful selection of the excitation wavelength and SPR resonant wavelength, which is determined by the materials that constitute the sensor, is essential for successful application.

#### 2.1.3. Phase Interrogation

A less common interrogation technique used in SPR fiber sensors is phase interrogation. The method relies on analyzing the phase difference between two light polarizations, p- and s-, at the resonance in the transmitted or back-reflected light [29,30,31]. This technique offers advantages of lower resolution and higher dynamic range when compared to other methods [31]. However, the requirement for more complex optical instruments and data processing methodologies result in limited applications for this interrogation technique in the context of fiber-based SPR sensors.

### 2.2. Performance Characteristics of SPR Fiber Sensors

The main characteristics that describe the performance of SPR fiber sensors are the refractive index sensitivity, resolution and detection limit. The refractive index sensitivity (*S*) is defined as a change in the monitored parameter, such as a resonant wavelength, intensity, or phase difference, with respect to the refractive index change [4]. Higher sensitivity is typically attributed to fibers made of a dielectric with lower refractive index. This fact is mainly due to the material dispersion as described by Homola [32]. Sensitivity analysis of plasmonic probes has shown that the sensitivity is strongly dependent on the optical properties of materials that constitute the sensor, such as the fiber material and metal film, rather than a particular sensor geometry [32,33]. In addition, the spectral sensitivity scales with the resonance wavelength.

While the sensitivity is often perceived as the most important performance parameter, another important characteristic is the sensor’s resolution. The resolution (*R*) is defined as the smallest shift in the monitored parameter, such as a wavelength, detectable by a sensing platform. It depends on the optical setup and parameters of the SPR spectral signature (i.e., Full Width at Half Maximum (*FWHM*) and Signal to Noise Ratio (*SNR*)) [34,35]. The resolution is also strongly influenced by the properties of the sensor materials (i.e., the metallic coating and fiber glass), interrogation and excitation methods, instrumentation, as well as the system noise [35].

Both, the sensitivity and resolution determine the most crucial performance characteristic—the Detection Limit (*DL*), which is the smallest change of the refractive index measurable. The detection limit is defined as the ratio between *R* and *S*, and, consequently, to improve the sensor’s *DL*, one would have to increase the sensitivity of the sensor, and/or reduce its resolution. The later can be achieved by either minimizing the *FWHM* of the SPR signature and/or increasing its *SNR*. However, there is often a trade-off between the sensitivity and spectral linewidth of the SPR response due to the properties of the sensor materials. It is difficult to establish a proper value of the resolution from the information provided in literature. As the resolution is proportional to the *FWHM*, it is more convenient to define a Figure of Merit (*FOM*), as the ratio between the *FWHM* and *S*, for a fair comparison between the different fiber-based SPR architectures. 

## 3. Plasmonic Fiber Sensor Designs

**Figure 2:**
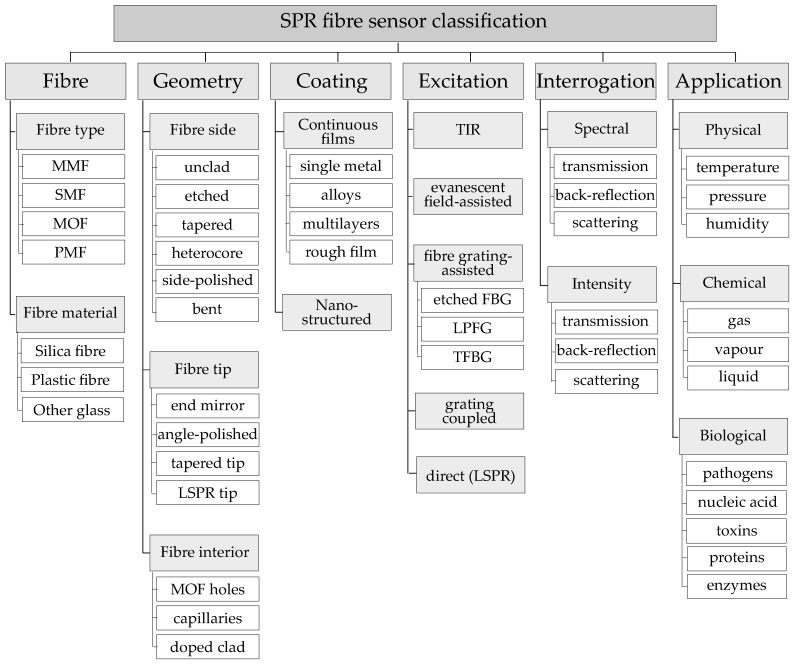
Classification of SPR fiber sensors. MMF: Multi Mode Fiber; SMF: Single Mode Fiber; MOF: Microstructured Optical Fiber; PMF: Polarization Maintaining Fiber; FBG: Fiber Bragg Grating; LPG: Long Period Fiber Gratings; TFBG: Tilted Fiber Bragg Gratings; LSPR: Localized Surface Plasmon Resonance.

Optical fibers are designed to guide light inside the fiber with as little loss as possible. Therefore, the construction of conventional optical fibers, such as the ones used for telecommunication, prevents guided modes from escaping into the surroundings, typically through the use of fiber claddings. To allow the guided fiber modes to interact with the plasmonic materials, the optical fiber geometry needs to be modified to suit SPR sensing applications. The flow chart in Figure 2 summarizes different fiber-based SPR sensor configurations, encompassing the variety of fiber geometries, plasmonic coatings, excitation and interrogation methods, as well as different applications.

Many fiber sensors are constructed using similar architectural principles as the plasmonic fiber sensors described below. For example, evanescent-field fiber sensors take advantage of the modes that extend beyond the boundary of the optical fiber. Fiber Bragg Grating (FBG) sensors rely on changes in properties of the grating with the change in the measured parameter, such as temperature for example. Fiber dip sensors use specialized coatings that modulate optical or physical properties as a response to a change in the measured parameter. However, plasmonic coatings significantly enhance light-matter interaction due to the strong electromagnetic field near the interface allowing more sensitive probes and enabling surface enhanced spectroscopy.

### 3.1. Optical Fiber Architectures

SPR fiber sensors can be categorized according to the type of optical fiber used as: Multimode Fiber (MMF), Single Mode Fiber (SMF), Microstructured Optical Fiber (MOF), Polarization Maintaining Fiber (PMF), or multiple-core fiber SPR sensors. Early fiber optic SPR sensors were mainly constructed from large core MMFs, where a small section of the polymer cladding along the fiber length was removed to expose the core for subsequent metal deposition, as shown in Figure 1. MMFs are among the most common fiber types used in SPR sensing due to their abundance and a relative simplicity of sensor fabrication. However, the large number of modes propagating inside an MMF causes broadening of the SPR spectral linewidth, resulting in decreased sensing performance. One approach to overcome this limitation is the reduction in the number of propagating modes that can satisfy the resonant condition. This could be achieved through the use of fewer mode fibers or SMFs, that typically have step- or graded-refractive index profile, a small core, and a low numerical aperture. SMF-based SPR probes remain the gold standard for the SPR fiber technology due to their low resolution. However, fabricating an SPR sensor from an SMF require additional fiber processing, such as polishing or tapering, as the claddings are typically made of glass. These requirements make the fiber probes extremely fragile, which eventually limits their applicability.

MOFs are becoming increasingly popular as fabrication techniques advance. They offer the convenience of reasonable mechanical strength, together with the ability to achieve desirable guiding properties through the design of the microstructure. Various MOF-based SPR sensors were explored theoretically [36], but only a handful have been experimentally realized [37]. These include a specialty MOF-based probe with metallized air holes [36], suspended core MOF sensor [38], as well as more recent configurations, such as a D-shaped hollow core MOF [39], a Photonic Crystal Fiber (PCF)-based SPR sensors [40,41], and an exposed core MOF SPR probe [33,37,42]. The major limitation for the use of the MOFs for SPR sensing is the cost of fiber fabrication and the complexity of depositing plasmonic materials on the interior structures of the MOF’s core.

#### 3.1.1. Fiber-Side SPR Sensors

**Figure 3:**
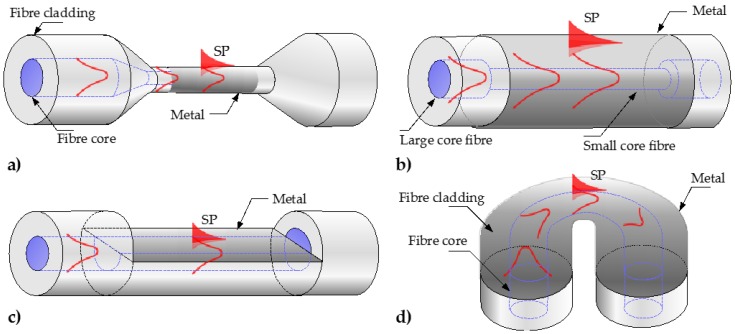
Schematics of geometry-modified optical fiber SPR sensors implemented on a side of an optical fiber: (**a**) Tapered fiber SPR probe; (**b**) Hetero-core structure; (**c**) D-shaped SPR probe; (**d**) U-shaped SPR probe.

Different techniques are used to produce sensing probes on a side of an optical fiber, which depend on the fiber type and fabrication method. These fiber-side SPR configurations range from unclad or etched optical fibers [43,44], to tapered fibers [45], hetero-core structures [19,46], side polished fibers [20,47,48], and bent fiber probes [49]. Figure 3 schematically shows the geometry-modified optical fiber SPR sensors implemented on a side of an optical fiber.

For a large core MMF where the cladding typically consists of a soft polymer layer, the core can be easily exposed by removing a section of the polymer layer [43,50,51]. However, if the cladding is made of glass, like in double-clad or graded-index fibers, etching can be performed to access the fiber core for metal deposition [52]. Alternatively, the fiber could be tapered over a short region along its length by heating a section of the fiber with a filament in a controlled manner [45]. In the tapered region of the fiber the evanescent field escapes through the cladding and interacts with the surroundings. The tapered region is then coated with a metal film for SPR sensing, as shown in Figure 3a. These structures predominantly benefit from having small cores, resulting in a narrow SPR feature, and therefore, an increased resolution. The refractive index sensitivity for tapered probes could also be enhanced through the stronger interaction of the propagating modes with the metallic coating, reaching up to 12,000 nm/RIU [45]. However, the fragility of the tapered probes makes them impractical for many applications.

To overcome this issue, other SPR fiber configurations, called hetero-core structures, have been proposed [16,19,46,53]. The configuration consists of two different types of fibers with mismatched core diameters, typically an SMF fiber spliced between two MMF fibers. The SMF region is coated with a metallic thin film for SPR sensing, as shown in Figure 3b. The mismatch between the cores causes guided modes to couple into the cladding of the SMF fiber where plasmonic interaction can occur. However, the sensitivity of the hetero-core SPR sensors (2000 nm/RIU [46]) is still lower than tapered fibers [45].

As an alternative to etching or tapering, side polishing has also been used to expose a section of the fiber core, resulting in a “D-shape” cross section, as illustrated in Figure 3c. To perform the polishing, a fiber is typically enclosed inside a resin block and polished off [54,55]. Wheel polishing setups have also been used to fabricated D-shaped fibers for SPR sensors [20]. Feasibility of MOFs as D-shaped SPR probes has also been theoretically investigated by many authors [39,47,48,56,57,58], but has not yielded any experimental demonstrations. Sensitivity of side-polished structures vary depending on fiber core size and material, with up to 4300 nm/RIU reported for an SMF-based side-polished SPR sensor [20]. Recently, an “eccentric core fiber” SPR probe, consisting of two SMFs with angle-polished tips spliced together was proposed and demonstrated by Liu et al. [59] showing the experimental sensitivity of up to 4738 nm/RIU. The ability to adjust the polishing angle allows for realization of sensing probes operating at specific wavelengths. This concept can be expanded for distributed sensing, owing different sensing regions can be produced along a single fiber.

Besides tapering, polishing or splicing different fibers together, bending offers the easiest way of extracting the light propagating inside the fiber core. When a fiber is bent beyond its critical radius, the propagating modes inside the core can couple into the cladding [49,60,61]. If the bent region is coated with a metal, a portion of this radiation can excite SPs. Bent fiber probes, also called “U-shaped” fibers, are usually fabricated from unclad large core MMFs or glass-clad fibers by heating a fiber section and forming a permanent U-shaped bend, as shown on Figure 3d. The sensitivity of bent SPR fiber sensors increases as the bend radius decreases, with theoretical values of up to 70,000 nm/RIU estimated for bend radius of 10 mm [62]. This predicted values are several times higher than for tapered probes, and around 25 times higher than for a straight sensing region implemented on the same fiber [62]. Feasibility of bent MOF SPR sensors have also been theoretically investigated for potential performance improvement, tunable by bending [63]. However, a recent experimental study, comparing bent and straight side-polished SPR probes revealed no significant improvement for the bent SPR fiber (bent radii of 10, 15 and 20 mm) compared with a straight side-polished one [64].

#### 3.1.2. Fiber-Tip SPR Sensors

**Figure 4:**
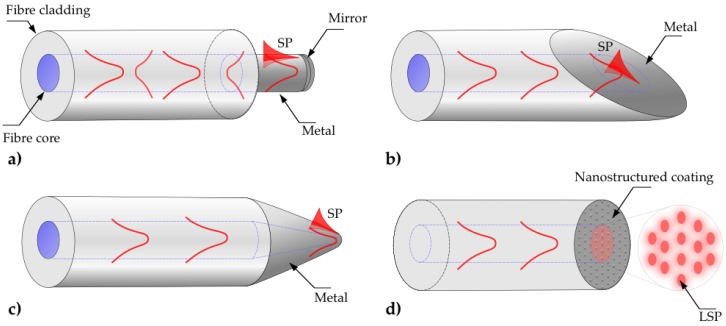
Schematics of geometry-modified optical fiber SPR sensors implemented on a tip of an optical fiber: (**a**) Flat fiber tip SPR probe with end mirror; (**b**) Angle polished flat fiber tip SPR sensor; (**c**) Tapered tip SPR probe; (**d**) LSPR fiber tip probe.

SPR fiber sensors can be implemented on, or near, a fiber tip, allowing for devices to be used as dip sensors. The simplest fiber tip SPR sensor consists of a plasmonic coating on a side of the fiber near its tip, which is coated with a metallic mirror, as depicted in Figure 4a. The dip SPR sensors are usually fabricated using MMFs and the interrogation is performed through analyzing light back-reflected from the mirrored fiber tip [24,65,66]. As this configuration is essentially the same as the unclad fiber SPR sensors described previously, the sensing performances are comparable to the fiber-side SPR sensors made of the same materials.

Alternatively, a dip sensor can be created by placing a plasmonic coating on the fiber tip. However, the incidence angles of the light propagating inside the fiber onto the straight cleaved end are typically small (near normal incidence), and below the range of SPR excitation angles. As a result, additional preparation of the fiber tip is required to increase the incidence angle. One of the existing techniques involves polishing the fiber end at a specific angle, thereby modifying the incidence onto the coated fiber tip, as shown in Figure 4b. The first fiber optic sensor with angle-polished fiber tip was proposed as early as 1993 [8], followed by further development [24,67,68,69], including polishing one fiber tip at different angles to acquire sensitivity to both, gas and liquid phases [66].

Recently, an interesting design of an angle-polished fiber tip SPR sensor that utilizes a specialty fiber with a twin core and the sensitivity of 5213 nm/RIU has been demonstrated [22]. An alternative approach to the angled-cleaved fiber tip is the modification of the fiber by tapering its tail until it forms an apex, similar to an Atomic Force Microscope tip. The tapered tip is then coated with a metallic thin film [52], as shown in Figure 4c. The performance of these SPR fiber sensors is comparable to the analogous side-tapered configurations, however, they are allegedly less fragile.

Another important subset of fiber-tip SPR sensors comprise fibers with a metallic pattern, supporting Localized Surface Plasmon Resonance (LSPR), fabricated on the fiber tip, as shown in Figure 4d. LSPR excitation is not restricted by the same constrains as propagating SPs, which require shallow incidence angles from a dielectric with refractive index higher than air, allowing for the excitation of localized SPs at normal incidence. As a result, LSPR sensors can be constructed directly on the straight cleaved fiber tip, which greatly simplifies the sensor architecture. With the recent advances in nanofabrication technologies, a variety of nanostructured surfaces have been used to pattern the fiber tip. These includes coating the fiber end with metal nanoparticle arrays [70,71], two-dimensional nanostructures [72], nano-hole arrays [73,74], as well as using a PCF with holes at the tip [75], or machining the metal coated fiber tip with sub-micron periodicity using Focused Ion Beam [76]. LSPR fiber tip sensors are an attractive platform due to the relative simplicity of the probe fabrication. However, LSPR is known for being less sensitive to refractive index changes when compared with sensors based on propagating plasmons.

#### 3.1.3. Specialty SPR Fiber Sensors

Instead of implementing the sensing region on the exterior of an optical fiber, plasmonic materials can be deposited on the fiber interior. Typically, this requires either MOFs with air holes or micro-capillaries. MOFs allow not only to take advantage of the fundamental guiding properties of these fibers, but also to use them as microfluidic channels for samples at the same time [77]. 

**Figure 5:**
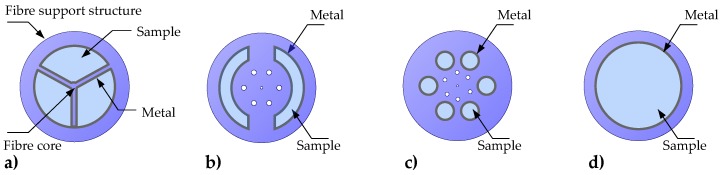
Examples of fiber interior SPR sensors: (**a**) Wagon-wheel fiber SPR sensor with triangular hole geometry [38]; (**b**) MOF fiber SPR sensor with crescent-shaped holes [36]; (**c**) PCF SPR sensor with circular holes [79]; (**d**) Microcapillary fiber SPR sensor geometry [80].

MOFs with various air cavity shapes coated with metal particles or films have been reported, including circular air holes [78] and crescent-shape holes [36], triangular shaped holes [38], and inner cavities of a PCF [79], as shown in Figure 5. Some specialty MOFs, such as an exposed core fiber, allow the deposition of plasmonic coatings without filling the fiber holes [37]. Nevertheless, practicality of the specialty MOF SPR sensors remains limited by the cost of fabrication of the fiber itself.

SPR sensor architectures with a silver coating deposited onto the inner microcapillary wall have recently emerged as an alternative to fiber-based SPR sensors [81,82,83]. Microcapillary-based SPR sensors can be highly advantageous as the capillary itself can be used as a microfluidic channel. However, this particular approach is only applicable for high refractive index liquids (typically *n* > 1.5) due to the requirement for confining the propagating light inside the capillary, which also results in larger sensitivity values of up to 6607 nm/RIU [80]. Despite these few successful experimental demonstrations, this area remains predominantly theoretical due to the practical difficulties of deposition of the plasmonic coatings on the inner cavities of the fiber microstructure, as well as by the cost of fabrication of the MOF itself. 

#### 3.1.4. Fiber Grating-Assisted and Grating-Coupled SPR Sensors

**Figure 6:**
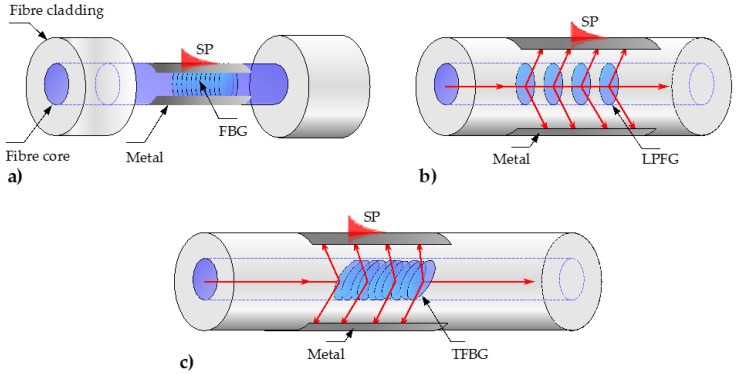
Schematics of the fiber grating-assisted SPR fiber sensors: (**a**) Etched Fiber Bragg Grating (FBG) SPR sensor; (**b**) Long Period Fiber Grating (LPFG) SPR sensor; (**c**) Tilted Fiber Bragg Grating (TFBG) SPR sensor.

The most common method of SPR excitation in optical fibers is Total Internal Reflection (TIR) where a proportion of the evanescent field that penetrates through the interface on ordinary total reflection can excite SPs. An additional increase of the evanescent field can be achieved by introducing inhomogeneity in the fiber core structure, such as tapering, hetero-core, bending, or side-polishing, as described in Section 3.1. A remarkable progress in SPR excitation has been achieved through the use of fiber gratings. A fiber grating is a periodic structure in the refractive index of the fiber core which generates a wavelength-specific dielectric mirror. A review of the fiber grating fabrication techniques can be found elsewhere [84]. Due to their mechanical strength and availability, fiber gratings became popular for construction of SPR sensors in recent years. Instead of modifying the fiber geometry to access the core-guided light, fiber gratings such as FBGs, Long Period Fiber Gratings (LPFGs) or Tilted Fiber Bragg Gratings (TFBGs) can be used to diffract a portion of the guided light from the core into the cladding, where it can interact with the plasmonic coating, as shown in Figure 6a–c, respectively. These fiber gratings, and especially the LPFGs and TFBGs, overcome the major limitation of geometry-modified SPR fiber sensors, enabling the plasmonic coating to be simply deposited onto the cladding. Several configurations of the LPFG-assisted SPR fiber sensors have been reported [23,85,86]. Recently, LPFGs have been proposed to be used in an SPR fiber sensor implemented by bending a fiber in a loop [84,87,88].

The refractive index sensitivity achieved with TFBG SPR sensors is generally moderate (400–1000 nm/RIU) [89] in comparison to TIR and evanescent field-assisted propagating plasmon-based sensors, which typically reaches up to several thousand nm/RIU. Despite that, due to the nature of TFBG SPR excitation, the spectrally well-defined cladding modes allow higher precision in determining the position of the SP resonant feature. This allows much higher detection accuracy and lower resolution, making TFBG SPR probes applicable for real-time biological sensing [90]. In addition, one of the most important features of the TFBG-assisted SPR sensors is the presence of the Bragg resonance which could be used as a reference control for compensation of temperature and optical power fluctuations [12]. It is important to differentiate between grating-assisted and grating-coupled SPR fiber sensors. As described above, in the grating-assisted sensors, the fiber grating inscribed in the core is used to out-couple the guided light into the cladding, where it interacts with the metal coating, thereby excites SPs. In grating-coupled SPR configurations, the grating is inscribed in the metallic coating and is used as a coupler to adjust the propagation constant of light incident to the metal [91]. As an alternative to the metal grating, periodic corrugations could be fabricated on the fiber surface or fiber tip, where metal is deposited over the dielectric grating [92].

### 3.2. Plasmonic Coatings

As the dielectric function of metals plays a crucial role in the plasmonic interaction, fundamental properties of the surface plasmons, and therefore, their performance parameters, are significantly affected by the metal coating. Typical materials that are used for plasmonic coatings are silver (Ag), gold (Au), aluminum (Al), and copper (Cu) [93]. The sensor performance, such as sensitivity, resolution, and dynamic range, is greatly influenced by the choice of the metal and the metallic coating morphology. Ag generally provides higher sensitivity due to the high magnitude of the real part of its dielectric constant and lower imaginary part [3], but owing to its chemical resilience, Au is more commonly used. Although, more exotic materials, such as indium tin oxide (ITO) [44,94] and graphene [40,95], have recently emerged as alternative plasmonic materials for optical fiber SPR sensors. The metallic thin film thickness is also known to influence the SPR sensor performance, with the optimal thickness of around 40–60 nm [4]. In addition to the metal type and its thickness, the film roughness can significantly affect the SPR signal. Smooth metal films are traditionally used for most SPR configurations due to higher propagation length of the SPs and, therefore, better performance. However, metallic coatings with high surface roughness can be used to induce SPR scattering caused by spatial variation of the dielectric constant of the film [96,97], which allows an alternative approach for SPR spectral interrogation [51]. Surface plasmons can be generated on metal coatings of various structures, such as conventional propagating surface plasmons on a single metal layer, alloys, or double-metal structures, as well as plasmonic nanostructures for LSPR and Long Range Surface Plasmons (LRSPs) on dielectric-metal structures.

#### 3.2.1. Continuous Metal Films: Propagating Plasmons

**Figure 7:**
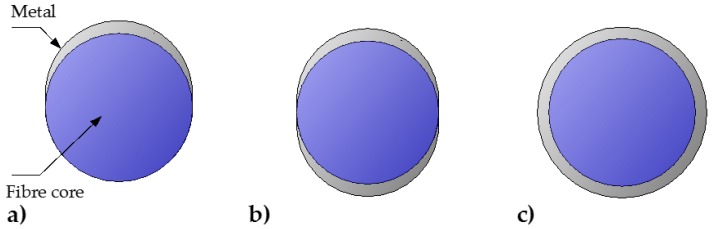
Cross sections of optical fiber core with common metal coating layouts: (**a**) Asymmetric one-sided coating; (**b**) Symmetric double-sided coating; (**c**) Symmetric cylindrical coating, typically produced by chemical deposition.

The most common plasmonic coating is a single-metal smooth continuous thin film deposited using physical deposition methods, such as thermal evaporation or sputtering. However, as physical deposition methods are intrinsically directional, a rotational apparatus is required to fabricate homogeneous coating onto the fiber, especially for fiber-side SPR sensors. Chemical deposition methods, such as chemical vapor deposition [98], or electroless plating [99], can easily achieve symmetric coatings around the fiber circumference [51] or inside MOFs [99]. Metallic coatings with varying thickness, such as single- or double-sided coatings, shown in Figure 7a,b respectively, were reported to result in multiple resonances [100], as well as an increase in the sensor’s dynamic range [21,26]. However, these layouts of the plasmonic coatings can influence the sensor performance by broadening the SPR spectral feature. In addition, stronger SPR signals are typically obtained with double-sided (Figure 7b) and cylindrical (Figure 7c) coatings [101].

Using rough metal coatings can provide some advantages despite the lower SPs propagation length. The scattered radiation can be used to infer the SPR signal [43,50,51], instead of performing transmission measurements, as it is done in fiber SPR sensors with smooth plasmonic coatings. In general, the larger spectral linewidth of the SPR response is associated with rough metal surfaces when compared to the smooth metal films [102]. However, amongst advantages of using metal coatings with high roughness are the lower reliance on the film thickness, and the ability to fabricate those coatings using simple wet chemistry techniques, such as electroless plating [51,99].

#### 3.2.2. Layered Coatings: Conventional, Long Range and Waveguide Coupled Plasmons

Instead of using a single metal or alloy as the propagating plasmons’ supporting material, coating of multiple metals can be designed to provide the desired performance, as well as to improve binding of the plasmonic coatings to a dielectric substrate. Bimetallic coatings consisting of silver and gold have been used to increase sensor performance, as well as to protect silver from oxidation [103,104]. Plasmonic films can also be stacked with dielectric or metallic layers to produce structures that modify SPR sensor characteristics. Dielectric layers (i.e., silica, magnesium fluoride, fluorinated polymers) deposited on top or between metal films have been used to improve sensing performances [26,105,106], tune the resonant wavelength [107], to generate LRSPR [82,108], or waveguide coupled plasmons [47], and as protective coatings [109]. 

#### 3.2.3. Nanostructured Coatings and Nanoparticles: Localized Plasmons

Nanostructured periodic and non-periodic plasmonic coatings, including metal particles of various shapes and sizes [110,111], metal nanoshells [112], nanostrips [113], nanorings [114], periodically arranged nanostructures [74], have been used to produce LSPR fiber optic sensors. Various methods to prepare this nano-patterned materials exist today which have been extensively described in literature [115]. The intrinsic refractive index sensitivity of localized SPs is a few orders of magnitude smaller than that of sensors based on SPs propagating on planar surfaces. Nevertheless, LSPR still provides some advantages such as lower cost, smaller sensing elements for high spatial resolution, and better surface sensitivity [116]. The SP field is more tightly concentrated at the vicinity of the sensor surface in the case of LSPR, so the decay length of LSPR is usually significantly smaller than that of SPs. This results in the higher surface sensitivity of localized plasmons, which can be beneficial for small particle detection [117]. In addition, the radiative nature of optical manifestation of localized surface plasmon excitation establishes the dominance of LSPR for surface enhanced optical phenomena in optical fibers [118], such as Surface Enhanced Raman Scattering (SERS), Surface Enhanced Resonant Raman Scattering (SERRS), and Metal Enhanced Fluorescence (MEF).

## 4. Applications

Intrinsically, an SPR sensor is a refractometer that only produces response to a change in the RI of the sensed medium. SPR fiber sensors have found applications as physical, chemical and biological sensors. However, additional elements, such as surface functionalization or physical modifications to the sensor surface, need to be incorporated in the architecture in order to generate refractive index change in response to the parameter to be determined. Besides, some environment change (e.g., temperature fluctuation) also leads to refractive index change, and thus SPR signal variation, which is especially significant in the case of biosensing. Either monitoring the reference or stabilizing environment could eliminate this effect to some extent from the background.

### 4.1. Physical SPR Fiber Sensors

SPR sensors have been used for the detection of physical quantities such as temperature, pressure, and humidity. As the SPR resonant condition is, in general, not highly sensitive to those quantities, specially designed functional coatings that translate change in these physical parameters into a change in the refractive index, need to be used. Thermal modulation of the refractive index of a material is described by its thermo-optic coefficient. Refractive index of the fiber core (i.e., typically fused silica) and plasmonic coating materials do not change significantly with temperature [119,120]. Therefore, temperature SPR fiber sensors are typically constructed using dielectric materials with a high-value thermo-optic coefficients deposited onto the sensor surface [121,122,123,124]. SPR fiber optic sensors for pressure detection have also been demonstrated. Here, an elastic polymer which refractive index depends on the applied pressure was used [125]. A theoretical design of an SPR fiber probe for pressure sensing has been proposed by Duarte et al. [126], where a U-shaped SPR probe was enclosed in a silicon rubber block. When pressure was applied to the block, the bend radius of the fiber probe changed, resulting in the change of the resonant condition. Similarly, SPR humidity sensors rely on functional materials whose refractive index depends on the number of water molecules adsorbed onto it. Typically, humidity sensitive polymeric over-layers in the proximity of a plasmonic coating are used to construct SPR-based fiber optic humidity sensors [44,127]. Change in humidity near the polymeric layer originates variations of the coating thickness, as well as its refractive index, causing a shift in the SPR condition. SPR dew sensors can detect temperature and humidity gradients in the adjacent environment by detecting changes of thickness of the water layers forming on the sensing surface [128].

### 4.2. Chemical SPR Fiber Sensors

SPR fiber sensors have also been exploited for chemical sensing in liquid or gas phases. The presence of certain chemicals is detected by either designing a functional coating, capable of specifically interacting with the chemical of interest, or direct change of the refractive index induced by the presence of the sensed target component in the sample. For example, pyrrole/chitosan composite coating was used to bind heavy metal ions onto Ag-ITO coating for detection of water contamination [129]. The detection of hydrogen peroxide using an SPR fiber sensor based on Ag film coated with Ag nanoparticle-doped polyvinyl alcohol nanocomposite has also been reported [130]. Other methods rely on dependence of dielectric’s refractive index on concentration of target chemicals, such as a water salinity sensor [131]. Several pH fiber optic LSPR sensors have also been demonstrated [132,133]. Gas sensing with SPR typically relies on metals (palladium, silver) or semiconducting metal oxide over-layers (indium tin oxide, tin dioxide, nickel oxide, zinc oxide, titanium dioxide) that change their electric permittivity when exposed to gaseous environments. Some example of gas SPR chemical fiber optic sensors include ammonia sensor [134], chlorine gas sensor [135], hydrogen SPR sensor [94,107,136], and hydrogen sulfide gas sensor [137].

### 4.3. Biological SPR Fiber Sensors

By far the largest application domain for SPR fiber sensors is biological sensing. This is due to the important advantages these sensors offer in comparison to other biosensing methods, such as the label free aspect, small volume of samples, quantitative real-time measurements, and possibility of in vivo and in-situ deployment. For an SPR response to occur in a biosensor, the sensing surface is functionalized with bioreceptors, which are designed to interact specifically with the analyte and produce a measurable change in its refractive index. Examples of bioreceptors include antibodies [138], aptamers [139], enzymatic bioreceptors, proteins, and nucleic acids. SPR fiber-based sensors have been applied for detection of pathogens, such as viruses [51,140], bacteria [141], biomarkers of medical conditions [43,50,142,143], study of DNA and DNA-protein interactions [144,145], and detection of bio-hazardous compounds [146,147,148]. However, even with its high sensitivity, SPR sensors cannot be successfully applied for a single molecule detection, unlike other optical resonance phenomena such as Whispering Gallery Mode [149,150], which typically exhibit much higher resolution. Moreover, the inherent drawback of biosensing with SPR, like any label-free method, lies in the intervening of the outcome of the results by non-specific binding, which can eventually result in false positives. However, due to the nature of the plasmonic coating (i.e., silver or gold), SPR and, therefore SPR fiber sensors, have a decisive advantage compared with other optical methods. Thiols are known to strongly interact with either Au or Ag and form self-assembled monolayers (SAMs), which can subsequently be used for the immobilization of antibodies [138], aptamers [139], or other relevant biomolecules. In addition, thiol-terminated polyethylene glycol SAMs are known for their ability to minimize or completely suppress non-specific binding in complex biological samples, such as serum [151,152].

## 5. Conclusions and Perspectives 

It is often difficult to compare performance of SPR sensing devices due to the large variety of SPR fiber sensor architectures. Table 1 provides a summary of recent experimental demonstrations of SPR fiber sensors with their performance characteristics (*S*, and *FWHM*) and a description of major aspects of their configurations. In addition, the ratio between the *FWHM* and sensitivity is presented as a figure of merit to evaluate what would be the best performing platform in terms of an equivalent detection limit. 

Looking at Table 1, it becomes obvious that, in general, LSPR-based fiber sensors are vastly outperformed by the standard SPR approach in both, sensitivity and *FWHM*. LSPR is known to have lower refractive index sensitivity compared with SPR and a broader *FWHM*, where both parameters primarily depend on the feature size of either the metallic nanoparticles or patterns. Consequently, the ratio between *FWHM* and sensitivity for LSPR-based fiber sensors is significantly lower than that for SPR-based ones. For SPR fiber sensors, the *FWHM*, and eventually the detection limit, can be improved by reducing the number of propagating modes that satisfy the coupling conditions with the SPs. Therefore, there is a clear trend, where SMF-based SPR sensors tend to exhibit a lower *FWHM* to sensitivity ratio compared with MMFs ones. Yet, even with SMF-based SPR sensors, the *FWHM* is still limited to 40–50 nm [45,47,54,55]. The addition of a TFBG on the SMF core allows for further reduction of the *FWHM* down to 5 nm [154,155], greatly improving the figure of merit. Silver and gold are largely dominant as plasmonic coating materials, although some more exotic ones have been implemented. However, there is no clear trend of what would be the ideal plasmonic coating as either silver or gold exhibit similar refractive index sensitivities when compared with other materials. Similarly, the fiber architecture, whether it is side-coated or tip-coated, does not significantly influence the performance, but allows for different applications (i.e., dip sensing for tip-coated fibers). Among the diverse interrogation methods, transmission or reflectivity measurements yield similar results, although heterodyne interferometry implemented on a D-type SPR fiber sensor looks like a promising approach for improving the refractive index sensitivity [156,157].

With the dozens of commercial SPR instruments based on a prism configuration available today, fiber-optic SPR probes experience a very slow uptake. This is mainly due to the increased complexity of fiber design and plasmonic coating fabrication on an optical fiber, resulting in higher cost of the consumable sensors. Moreover, the necessity of manipulation and optical alignment of small fiber probes by the end-user makes the sensing probes even more expensive. However, SPR fiber sensors provide several important benefits over the standard prism configurations. Although, a considerable range of SPR fiber sensor designs have been proposed and demonstrated, there is significant scope for the development of improved SPR systems suitable for a range of applications. 

The aspects of development that need to be addressed for the fiber optic SPR sensors to be commercially feasible include but are not limited to engineering miniature sensing probes, improved microfluidic systems, low cost manufacturing techniques, as well as improved sensing performance, such as sensitivity and detection limit. Yet, some aspects of SPR that can significantly improve the sensing performance have not been demonstrated in fiber platforms. A key example is LRSPR, where only a few demonstrations involving optical waveguides and fibers have been reported [82,158]. LRSPR could be highly beneficial for biosensing applications, especially for large macromolecules, viruses, bacteria and other cells, whose size is beyond the standard penetration depth of SPs. LRSPR with its superior penetration depth compared with SPs would allow probing of entire macromolecules. In addition, it has been reported that the LRSPR refractometric sensing approach can achieve higher refractive index sensitivity [159], and holds the record detection limit of 2 × 10^−8^ RIU in a prism configuration [160]. Combining the LRSPR method within fiber sensing architectures, and taking advantage of fewer mode fibers for optimizing both, sensitivity and resolution, could lead to the development of new highly performing refractometric sensing approaches.

## Figures and Tables

**Table 1:** Summary and characteristics of recent experimental demonstrations of SPR fiber optic sensors.

Fiber	Coating	Excitation	Interrogation	S_λ_ nm/RIU	FWHM nm	FWHM/S_λ_ RIU	Reference
SMF Fiber tip	Au NPs ^4^	direct (LSPR)	spectral, trans.	196	ca. 70	3.6 × 10^−1^	[71]
MMF Side etched	Au NPs ^3^	TIR (LSPR)	intensity, trans.	ca. 500	ca. 50–150	3 × 10^−1^–10^−1^	[153]
SMF Fiber tip	Au holes ^4^	direct (LSPR)	spectral, back-reflect.	755	ca. 200	2.6 × 10^−1^	[73]
MMF Fiber tip	Ag NPs ^3^	Direct (LSPR)	spectral, back-reflect.	387	ca. 70–100	2.5 × 10^−1^–1.6 × 10^−1^	[111]
Microcapillary	Ag film	TIR, from sample	spectral, trans.	1000	ca. 200	2 × 10^−1^	[81]
MMF Flat & taper. tip	Au holes ^4^	direct (LSPR)	spectral, trans.	533	ca. 80	1.5 × 10^−1^	[76]
MMF Fiber tip	Au film ^1^	TIR	spectral, back-refl.	1433	ca. 150	10^−1^	[21]
MMF, GI SMF Heterocore	Ag film	ev.-field ass. TIR	spectral, transmission	1500–2100	ca.150–200	10^−1^–7 × 10^−2^	[46]
GI MMF Side-polished	Au film	ev.-field ass. TIR	spectral, trans.	1570	130	8.2 × 10^−2^	[48]
MMF Fiber tip	Au film ^2^	TIR	spectral, back-reflect.	1557	ca. 80	5.1 × 10^−2^	[65]
Plastic MMF Side polish, bent	Au film	ev.-field ass. TIR	spectral, trans.	1654–2978	70	4.2 × 10^−2^–2.3 × 10^−2^	[64]
Microcapillary	Ag/dielectric	TIR LRSPR	spectral, trans.	2000–6600	50–250	1.2 × 10^−1^–7.5 × 10^−3^	[82]
MMF Angle-polished tip	Au film	TIR	spectral, back-reflect.	ca. 2650	ca. 100–150	5.6 × 10^−2^–3.7 × 10^−2^	[66]
SMF Etched tip, side etched	Au film/TiO_2_	ev-.field ass. TIR	spectral, back-reflect.	3800–5100	ca. 150–200	5.2 × 10^−2^–2.9 × 10^−2^	[52]
MMF MOF Fiber side	Ag rough film ^2^	TIR	spectral, scatter.	1753	75	4 × 10^−2^	[37]
SMF Side-polished	Ag film	TIR	spectral, trans.	1523–4365	40–90	2.6 × 10^−2^–9 × 10^−3^	[20]
SMF, twin core Angle tip & mirror	Au film	TIR	spectral, back-reflect.	5213	ca. 100	1.9 × 10^−2^	[22]
MMF Side-polished	Au/ZnS-SiO_2_/Au ^1^	ev.-field ass. TIR	spectral, trans. (LSPR)	2200	40	1.8 × 10^−2^	[47]
SMF Side-polished	Cr/Au/Ta_2_O_5_^1^	TIR	spec.& inten., trans.	3300	ca. 50	1.2 × 10^−2^	[54,55]
SMF Side tapered	Au film ^1^	ev.-field ass. TIR	spectral, trans.	ca. 25000	ca. 150	6 × 10^−3^	[100]
SMF Side tapered	Al/InN bi-layer ^1^	ev.-field ass. TIR	spectral, trans	11,800	ca. 50	4.2 × 10^−3^	[45]
SMF Fiber side	Ag film ^1^	TFBG-assisted	spectral, trans.	550–673	5	7.4 × 10^−3^–9 × 10^−4^	[154,155]

^1^ Asymmetric coating; ^2^ Symmetric coating; ^3^ Non periodic structure; ^4^ Periodic structure.

## Abbreviations

The following abbreviations are used in this manuscript:

DL	Detection Limit
FBG	Fiber Bragg Grating
FOM	Figure of Merit
FWHM	Full Width at Half Maximum
GI	Graded Index
ITO	Indium Tin Oxide
LPFG	Long Period Fiber Grating
LRSPR	Long Range Surface Plasmon Resonance
LSPR	Localized Surface Plasmon Resonance
MEF	Metal Enhanced Fluorescence
MMF	Multi-Mode Fiber
MOF	Microstructured Optical Fiber
PCF	Photonic Crystal Fiber
PMF	Polarization Maintaining Fiber
SERRS	Surface-Enhanced Resonance Raman Scattering
SERS	Surface-Enhanced Raman Scattering
SMF	Single Mode Fiber
SNR	Signal to Noise Ratio
SP	Surface Plasmon
SPR	Surface Plasmon Resonance
TIR	Total Internal Reflection
